# Prevalence of prediabetes in patients with metabolic risk

**DOI:** 10.1590/S1516-31802011000500004

**Published:** 2011-09-01

**Authors:** Lívia Nascimento de Matos, Guilherme de Vieira Giorelli, Amir Saado, Cristiane Bitencourt Dias

**Affiliations:** I MD. Postgraduate Student, Department of Internal Medicine, Institute for Medical Treatment, Hospital do Servidor Público Estadual de São Paulo – Francisco Morato de Oliveira, São Paulo, Brazil.; II MD, PhD. Attending Physician, Department of Internal Medicine, Institute for Medical Treatment, Hospital do Servidor Público Estadual de São Paulo – Francisco Morato de Oliveira, São Paulo, Brazil.

**Keywords:** Glucose intolerance, Glucose tolerance test, Diabetes mellitus, Prediabetic state, Hyperglycemia, Intolerância à glucose, Teste de tolerância a glucose, Diabetes mellitus, Estado pré-diabético, Hiperglicemia

## Abstract

**CONTEXT AND OBJECTIVE:**

Early diagnosis of prediabetes should be done to avoid complications relating to diabetes mellitus (DM). The aim here was to assess the prevalence of prediabetes among individuals at high risk of developing DM, and to seek variables relating to glucose intolerance (GI) among individuals with normal fasting plasma glucose (FPG).

**DESIGN AND SETTING:**

Cross-sectional study at Hospital do Servidor Público Estadual, São Paulo.

**METHODS:**

The FPG and glucose tolerance test (GTT) were analyzed, from which the subjects were divided as follows: group 1 (FPG and GTT both normal), group 2 (normal FPG but abnormal GTT), group 3 (abnormal FPG but normal GTT), and group 4 (FPG and GTT both abnormal). The subjects’ clinical, laboratory and anthropometric profile was determined.

**RESULTS:**

138 subjects were studied: 44 in group 1, 11 in group 2, 33 in group 3 and 50 in group 4. The prevalence of prediabetes was 68.0%. Group 4 individuals were older than group 1 individuals [69.0 (55.5-74.0) versus 58.9 ± 11.8 years; P < 0.05], with greater prevalence of risk conditions for DM [5.0 (4.0-5.0) versus 4.0 (3.0-5.0); P < 0.05]. Among individuals with normal FPG, GI prevalence was 20.0%. No variables analyzed correlated with GTT.

**CONCLUSION:**

The prevalence of prediabetes was 68.0%, and 20.0% of subjects with normal FPG had GI. Although some anthropometric, clinical and laboratory variables have been correlated with DM and prediabetes, none, except for GTT, was able to screen for GI among subjects with normal FPG in the present study.

## RESUMO

**CONTEXTO E OBJETIVO:**

Deve-se diagnosticar o estado pré-diabético precocemente para evitar as complicações do diabetes mellitus (DM). Objetiva-se avaliar a prevalência de estado pré-diabético entre indivíduos sob risco de desenvolvimento de DM, buscando variáveis relacionadas à intolerância à glicose (IG) naqueles com glicemia de jejum (GJ) normal.

**TIPO DE ESTUDO E LOCAL:**

Estudo transversal no Hospital do Servidor Público Estadual, São Paulo.

**MÉTODOs:**

Analisou-se GJ e teste oral de tolerância a glicose (GTT), que determinaram a seguinte divisão: grupo 1 (GJ e GTT normais), grupo 2 (GJ normal e GTT alterado), grupo 3 (GJ alterada e GTT normal) e grupo 4 (GJ e GTT alterados). Determinou-se o perfil clínico, laboratorial e antropométrico dos indivíduos estudados.

**RESULTADOS:**

Estudaram-se 138 indivíduos: 44 no grupo 1, 11 no grupo 2, 33 no grupo 3 e 50 no grupo 4. A prevalência de estado pré-diabético foi 68,0%. Indivíduos do grupo 4, em relação ao grupo 1, demonstraram idade mais avançada [69,0 (55,5-74,0) *versus* 58,9 ± 11,8 anos, P < 0,05] e maior prevalência de condições de risco para o DM [5,0 (4,0-5,0) *versus* 4,0 (3,0-5,0), P < 0,05]. Dentre aqueles com GJ normal, 20,0% apresentaram IG. Nenhuma variável se correlacionou com o GTT.

**CONCLUSÃO:**

A prevalência de estado pré-diabético foi 68,0%; 20,0% daqueles com GJ normal apresentaram IG. Apesar de algumas variáveis clínicas, antropométricas e laboratoriais estarem descritas como relacionadas ao DM e ao estado pré-diabético, no presente estudo, nenhuma, à exceção do GTT, foi capaz de rastrear a presença de IG entre aqueles com GJ normal.

## INTRODUCTION

Diabetes mellitus is a metabolic disorder characterized by high plasma glucose levels, resulting from lower insulin secretion, resistance to its peripheral action, or both.^1^ On the other hand, prediabetes may be defined as a state of abnormal fasting plasma glucose, glucose intolerance, or both.^1^

Over recent decades, this condition has come to be considered a worldwide pandemic. Data from the World Health Organization (WHO) indicate that the prevalence of diabetes mellitus is 2.8% among the world population over 20 years of age.^2^ Estimates from WHO have predicted that the worldwide prevalence will reach 4.4% by 2030, among aging individuals. In absolute numbers, this represents an increase from 171 million diabetic adults in 2000 to approximately 366 million around the world in 2030.^2^ These data may still be an underestimate, since the projections were made assuming that the overweight and obesity levels would remain stable among the world population over the coming decades.^2^ Brazil appears in eighth highest position out of 191 countries in relation to the ranking of diabetes mellitus rates among WHO members.^2^

However, many individuals have unknown diabetic or prediabetic metabolic abnormalities and live with high plasma glucose levels (either fasting or postprandial) for many years. Such levels may lead to establishment of tissue damage even before the classical signs and symptoms of this condition have become clinically established (polyuria, polydipsia, weight loss with or without polyphagia, and blurred vision). This has been recognized as increasing the risk of developing renal, cardiac, neurological, ophthalmological, macrovascular and microvascular complications, as well as infectious diseases.^1,3^ At the time when type II diabetes mellitus is diagnosed, some individuals already present some of those complications in laboratory tests.^2^ During this asymptomatic period, abnormal carbohydrate metabolism can be demonstrated through assessment of plasma glucose levels after 8-12 hours of overnight fasting (fasting plasma glucose, FPG) or through the glucose tolerance test (GTT), in which 75 g of glucose is ingested and the plasma glucose level is measured 120 minutes later.^1^ Plasma glucose levels may fluctuate between physiological and pathological levels among diabetics, depending on the extent of the underlying metabolic disorder, since the same clinical condition could lead to abnormalities only in postprandial glycemia, while FPG is normal, and vice versa.^1^ For such patients, interventions like weight loss, physical activity and use of oral hypoglycemic agents may lead to adequate glycemic control.^1^

The following conditions have been considered to increase the risk of developing diabetes mellitus: hypertension; overweight and obesity, defined as body mass index (BMI) ≥ 25 kg/m^2^; large waist circumference; first-degree kinship with diabetics; Asian, Hispanic or African-American ethnicity; mothers of large-for-gestational-age newborns or who presented gestational diabetes mellitus; fasting serum high-density lipoprotein (HDL) cholesterol < 35 mg/dl; and triglycerides > 250 mg/dl.^4^

Anthropometric indicators of central obesity have been associated with insulin resistance, demonstrated by high homeostasis model assessment of insulin resistance index (HOMA-IR); glucose intolerance and cardiovascular events, such as the conicity index^5^ and waist-to-height ratio (WHtR);^6^ as well as indicators of fat distribution body, such as waist-to-hip-ratio (WHR).^7,8^

Rosenbaum et al., who studied populations at high risk of developing metabolic disease, observed that glucose intolerance had an independent effect on endothelial dysfunction, which was characterized in their study by the presence of albuminuria.^9^ Furthermore, glucose intolerance is a condition of increased risk of developing diabetes mellitus.^10^ However, progression to diabetes mellitus is not inevitable,^2^ which suggests that early diagnosis of this clinical condition is desirable, such that prophylactic interventions can be adopted.

In Brazil, the prevalence of diabetes mellitus was estimated to be 7.6% and glucose intolerance was found to be 7.8%, 25 years ago, according to the Brazilian Multicenter Study, conducted between 1986 and 1988.^9^ Another study conducted in São Paulo between 1996 and 1997 showed higher estimated prevalence of diabetes mellitus (12.1%) and around the same glucose intolerance rate (7.7%).^10^ Another Brazilian study showed estimated prevalence of glucose intolerance of 14.7% among patients at high risk of metabolic syndrome in 1998.^11^

We believe that studies among non-diabetic populations that are at higher risk of developing diabetes mellitus are important for establishing preventive strategies directed towards this population.

## OBJECTIVES

This study aimed to assess the prevalence of glucose intolerance and abnormal FPG among outpatients presenting at least one of the conditions known to increase metabolic risk. These patients were followed in a tertiary care hospital in the city of São Paulo, in order to provide current data that could be of value for implementing future preventive strategies, and also to determine the anthropometric profile of these individuals. We hypothesized that a population at high risk of developing diabetes mellitus would show a prevalence of prediabetic states of at least 25%. We also hypothesized that, among the individuals with glucose intolerance, at least 10% would present normal FPG. Thus, the secondary objective of the study was to find anthropometric characteristics that might be related to glucose intolerance among individuals with normal FPG, and to determine the same correlations among the total study sample.

## METHODS

### Study design

We conducted a cross-sectional study in which we analyzed data from outpatients followed between July 2008 and December 2009 in the Department of Internal Medicine of the Institute for Medical Treatment, Hospital do Servidor Público Estadual de São Paulo – Francisco Morato de Oliveira, São Paulo, Brazil. Most of the individuals included were undergoing outpatient follow-up treatment for hypertension and/or dyslipidemia. The study was approved by the research ethics committee of the same hospital (protocol number 0010.338.000-08) and the research subjects gave their written informed consent.

### Inclusion criteria

The study included individuals who had at least one of the following conditions relating to higher risk of developing diabetes mellitus: hypertension; BMI ≥ 25 kg/m^2^; waist circumference > 80 cm for women and > 94 cm for men; first-degree kinship with diabetics; mothers of large-for-gestational-age newborns or who presented gestational diabetes mellitus; fasting serum HDL-cholesterol < 35 mg/dl; and triglycerides > 250 mg/dl. Since the Brazilian population is one of the most mixed in the world, ethnic groups were not considered as inclusion criteria alone.

### Exclusion criteria

The following were exclusion criteria: prior diagnosis of diabetes mellitus or a prediabetic state; use of oral hypoglycemic agents or insulin; use of drugs that would interfere with glucose and insulin metabolism, such as angiotensin-converting enzyme inhibitors, angiotensin receptor blockers and thiazide diuretics; use of drugs that would interfere with the serum levels of HDL-cholesterol and triglycerides; and use of any pharmacological drugs in order to treat obesity. Changes in lifestyle such as treatment for obesity, dyslipidemia, metabolic syndrome or any pathological condition did not constitute exclusion criteria. The only lipid-lowering drugs that were used by some research subjects were statins. Previous diagnoses of diabetes mellitus were defined as plasma glucose > 200 mg/dl at any time, FPG > 125 mg/dl, or GTT ≥ 200 mg/dl. Two results were necessary for the diagnosis when the patient was asymptomatic.

### Sample size calculation and study population

The study sample was calculated by estimating a prevalence of prediabetic state of 25%; the null hypothesis was defined as 15% prevalence. The alpha and beta errors were set at 0.05 and 0.20, respectively. Through the test sample calculation for single proportions, the minimum sample size required was estimated to be 106 individuals.

**Figure 1.**
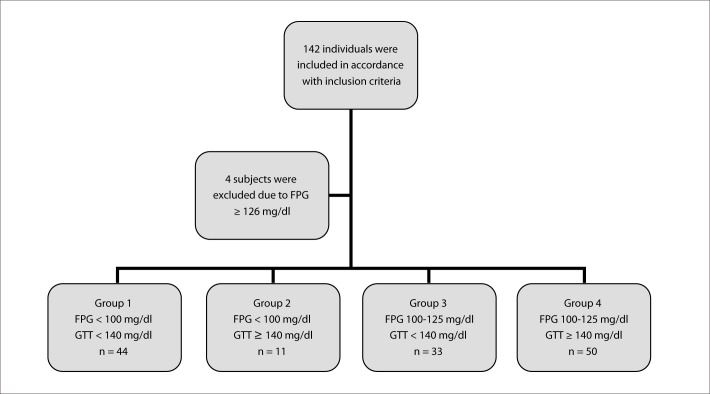
Flowchart showing the initial study population, the excluded subjects and the division into groups according to fasting plasma glucose (FPG) and glucose tolerance test (GTT) values.

Initially, the study sample included 142 individuals, who were mostly followed because of hypertension and dyslipidemia. Of these, four (2.8%) were excluded because of FPG ≥ 126 mg/dl, thus leaving 138 subjects. These were divided into groups (1, 2, 3 and 4) according to their FPG and GTT, as showed in the flowchart of Figure 1.

### Anthropometric measurements

All the data were evaluated by physicians trained in the current techniques recommended for each anthropometric measurement. We assessed weight and height while the subjects were wearing light clothes, using the techniques proposed by Jelliffe;^12^ waist circumference while the patients were standing, at the end of exhalation, at the midpoint between the lower costal border and the top of the iliac crest, using an inelastic tape in a horizontal position;^8^ and hip circumference, at the level of the greater trochanter,^13,14^ in order to calculate the WHR.^8,13,14^ We calculated the BMI, WHR, WHtR and conicity index. The formulas used to calculate the indices studied were as follows:

$$\begin{array}{l} \text{Body mass }{\text{index}}^{8}=\frac{\text{weight}\left(\text{kg}\right)}{{\text{height}}^{2}\left(\text{m}\right)}\\ \text{Waist}-\text{to}-{\text{hip ratio}}^{13}=\frac{\text{waist circumference }(\text{cm})}{\text{hip circumference }(\text{cm})}\\ \text{Waist}-\text{to}-{\text{height ratio}}^{6}=\frac{\text{waist circumference }(\text{cm})}{\text{height }(\text{cm})}\\ {\text{Conicity index}}^{\text{5}}=\frac{\text{waist circumference }(\text{m})}{0.109\times \frac{\sqrt{\text{weight }(\text{kg})}}{\text{height }(\text{m})}} \end{array}$$

### Clinical evaluation

We conducted a medical consultation covering the subject's history and focusing on asking about symptoms relating to diabetes mellitus, especially polyuria, polydipsia and weight loss with or without polyphagia. We assessed the research subjects’ blood pressure, presence of diagnoses of hypertension and dyslipidemia and use of any antihypertensive, lipid-lowering or other drugs.

We assessed blood pressure after the subjects had spent five minutes resting in a seated position, in a calm and warm environment, in both arms. The research subjects were encouraged to empty the bladder before the medical consultation, and were instructed not to eat or drink any products containing caffeine, or to smoke cigarettes during the two-hour period prior to the medical evaluation, in accordance with the Fifth Brazilian Guidelines on High Blood Pressure.^13^ Hypertension was defined as present when the blood pressure level was ≥ 140 x 90 mmHg on two different occasions or when antihypertensive drugs were being used, regardless of blood pressure levels.^13^

Diagnoses of dyslipidemia were evaluated in accordance with the laboratory criteria established in the Fourth Brazilian Guidelines on Dyslipidemia and Atherosclerosis Prevention,^8^ or were established if lipid-lowering medications were being used, regardless of the serum lipoprotein cholesterol and triglyceride levels.^8^

### Laboratory analysis

Blood samples were collected from the research subjects after they had spent 12 hours fasting (overnight) and after five minutes resting in a seated position. This was done by means of antecubital venous puncture. All the blood samples were analyzed by the same team in the same laboratory, using the same kits supplied by the same manufacturer. The GTT was performed using the blood samples collected after fasting and again, 120 minutes after ingestion of 75 g of glucose.

The research subjects underwent FPG and GTT analysis. They were instructed to adhere to a high-carbohydrate diet for three days prior to the GTT; not to use any laxative on the day before the test; and not to do any physical exertion just before the test. If individuals presented diarrhea during the 48-hour period preceding the GTT, it was scheduled for another day. Individuals were also instructed to avoid walking and they were not allowed to smoke throughout the test; ingestion of food of any kind was also prohibited during the test. The plasma glucose level was determined using an enzymatic method.

We evaluated the plasma insulin levels after the 12 hours of overnight fasting. These were determined using the immunometric method in a two-sided solid-phase chemiluminescent assay (Immulite 2000, Siemens^TM^). This assay shows high agreement levels between assays and within assays, according to information provided by the manufacturer. Plasma uric acid levels were determined using the enzymatic colorimetric method; plasma creatinine using the kinetic colorimetric method; plasma triglycerides using the enzymatic colorimetric method; plasma total cholesterol using the colorimetric method; and plasma HDL cholesterol using the enzymatic colorimetric method. Plasma LDL cholesterol was calculated using the Friedewald formula, as follows:

$${\text{LDL cholesterol}}^{8}=\text{total cholesterol}-\text{HDL cholesterol}-\text{triglycerides}/5.$$

We also evaluated microalbuminuria (μg/min) using the chemiluminescence method, in samples of 24-hour urine (data not presented).

FPG was considered to be normal when it was ≤ 99 mg/dl, and abnormal when it was between 100 and 125 mg/dl.^1^ GTT was considered to be normal when it was ≤ 139 mg/dl, and abnormal when it was ≥ 140 mg/dl.^1,15^ According to the World Health Organization criteria,^15^ GTT ≥ 200 mg/dl is regarded as diagnostic of diabetes mellitus in cases of postprandial hyperglycemia alone, but because this study aimed to investigate occurrences of glucose metabolism abnormalities among patients with normal FPG, we considered all values of GTT ≥ 140 mg/dl to be abnormal GTT.

### Statistical analysis

Statistical analyses were performed using the Medcalc software, version 11.1. The statistical significance level was set at P < 0.05.

Continuous variables were expressed as the mean ± standard deviation or median with interquartile range, for the variables with and without normal distribution, respectively. Categorical variables were expressed as percentages.

Differences between groups relating to categorical variables were determined using the chi-square test. For continuous variables showing normal distribution, analysis of variance with Tukey's post-hoc test was used, and Pearson's correlation coefficient was calculated. For those that did not show normal distribution, Kruskal-Wallis with Dunn's post-hoc test was performed, and Spearman's correlation coefficient was used. The receiver operating characteristic (ROC) curve was obtained, and the area under the curve (AUC) was calculated, with the 95% confidence interval.^16^ The sensitivity and specificity of the diagnosis of glucose intolerance, the anthropometric data and the FPG and HOMA-IR values were calculated for each cutoff point in the sample. The cutoff point with highest sum between sensitivity and specificity was chosen in order to optimize the relationship between those two parameters.^17^

## RESULTS

We analyzed the clinical, laboratory and anthropometric data of 138 individuals, whose general characteristics are presented in Table 1. It can be emphasized that metabolic risk conditions were highly prevalent in this population, and that the biggest three risk factors were obesity, high blood pressure and dyslipidemia.

**Table 1.** General characteristics of the population studied (n = 138)

Variables	Values
Male (n/%)	48/34.7
Female (n/%)	90/65.2
Age (years)	63.0 (54.5-71.5)
Weight (kg)	73.5 (67-85)
Height (m)	1.5 (1.5-1.6)
Body mass index (kg/m^2^)	28.8 (26.3-33.1)
Waist circumference (cm)	99.6 ± 12.2
Hip circumference (cm)	104.0 (99.0-113.0)
Waist-to-hip ratio	0.9 (0.8-0.9)
Waist-to-height ratio	0.6 (0.6-0.7)
Conicity index	1.3 (1.3-1.4)
High blood pressure (n/%)	92/66.6
Dyslipidemia (n/%)	90/65.2
Systolic blood pressure (mmHg)	130.0 (120.0-150.0)
Diastolic blood pressure (mmHg)	80.0 (80.0-90.0)
Number of risk conditions	4.0 (3.0-5.0)
Plasma creatinine (mg/dl)	0.9 (0.8-1)
Total cholesterol (mg/dl)	203.1 ± 39.7
HDL-cholesterol (mg/dl)	51.2 ± 12.8
LDL-cholesterol (mg/dl)	119.0 (97.5-147.0)
Triglycerides (mg/dl)	137.0 (88.5-166.0)
Uric acid (mg/dl)	6.1 ± 1.5
Insulin (mUI/ml)	8.2 (4.2-17.3)
HOMA-IR	1.9 (0.8-4.0)

Variables presented as mean standard deviation, median (interquartile range), or percentage. HDL = high-density lipoprotein; LDL = low-density lipoprotein; HOMA-IR = homeostasis model assessment of insulin resistance.

The population studied was divided into groups as follows: 44 individuals in group 1 (31.9%), who demonstrated normal levels both for FPG and for GTT (91.3 ± 5.5 mg/dl and 98.9 ± 22.1 mg/dl, respectively); 11 individuals in group 2 (8.0%), who demonstrated normal FPG and elevated GTT [92.4 ± 6.9 mg/dl and 149.0 (142.0-214.0) mg/dl, respectively]; 33 subjects in group 3 (23.9%), who presented abnormal FPG and normal GTT levels [107.0 (103.5 – 110.0) mg/dl and 110.5 ± 18.9 mg/dl, respectively]; and 50 subjects in group 4 (36.2%), who demonstrated high levels both for FPG and for GTT [108.0 ± 10.0 mg/dl and 158.5 (145.0-189.5) mg/dl, respectively].

The prevalence of a prediabetic state was 68.1% (Groups 2, 3 and 4) in the sample studied. Among the individuals who had a prediabetic state diagnosed during the study period, we found that 11.7% had glucose intolerance with normal FPG, 35.1% had abnormal FPG alone, and 53.2% had both glucose intolerance and abnormal FPG. Among the 61 individuals who had glucose intolerance diagnosed (groups 2 and 4), 11 (18.0%) demonstrated normal FPG. On the other hand, among the 55 individuals who had normal FPG, 11 (20.0%) demonstrated glucose intolerance or diabetes mellitus in relation to postprandial hyperglycemia alone.

In the comparisons between these groups, there were no differences regarding sex, BMI, waist circumference, WHR or prevalence of high blood pressure. However, in relation to individuals whose FPG and GTT were both normal (group 1), patients with abnormal FPG and elevated GTT (group 4) were older [69.0 (55.5-74.0) years versus 58.9 ± 11.8 years; P < 0.05], demonstrated higher number of risk conditions [5.0 (4.0-5.0) versus 4.0 (3.0-5.0); P < 0.05], higher plasma levels of uric acid (6.5 ± 1.6 mg/dl versus 5.5 ± 1.3 mg/dl; P < 0.05), lower prevalence of dyslipidemia (38.0% versus 61.3%; P < 0.001), lower plasma levels of LDL cholesterol (112.9 ± 35.8 mg/dl versus 134.4 ± 13.5 mg/dl; P < 0.05) and higher HOMA-IR index [2.6 (1.1-4.1) versus 1.1 (0.4-2.0); P < 0.05]. In relation to group 3, the individuals in group 4 demonstrated lower plasma levels of LDL cholesterol (112.9 ± 35.8 mg/dl versus 133.9 ± 33.6 mg/dl; P < 0.05). In relation to group 1, group 3 presented higher HOMA-IR index [5.9 ± 6.3 versus 1.1 (0.4 – 2.0); P < 0.05]. The comparisons between group data are shown in Table 2.

**Table 2.** Clinical, anthropometric and laboratory characteristics of each group and comparisons between groups 1, 2, 3 and 4

Variables	Group 1 (n = 44)	Group 2 (n = 11)	Group 3 (n = 33)	Group 4 (n = 50)	P
Male (n)	12	3	15	12	ns
Age (years)	58.9 ± 11.8	61.9 ± 20.7	59.7 ± 11.5	69.0 (55.5-74.0)	< 0.05*
Body mass index (kg/m^2^)	29.0 ± 5.7	30.4 ± 5.0	29.9 ± 4.8	29.9 ± 5.4	ns
Waist circumference (cm)	97.9 ± 13.4	95.0 (93.0-110.0)	100.7 ± 11.9	100.4 ± 12.1	ns
Hip circumference (cm)	103.0 (96.0-110.0)	105.1 ± 12.2	107.1 ± 9.6	104.5 (100.0-113.5)	ns
Waist-to-hip ratio	0.9 (0.9-1.0)	0.9 ± 0.1	0.9 (0.9-1.0)	1.0 (0.9-1.0)	ns
Waist-to-height ratio	0.6 ± 0.1	0.6 ± 0.1	0.6 (0.6-0.7)	0.7 ± 0.1	ns
Conicity index	1.4 (1.3-1.4)	1.3 (1.3-1.4)	1.3 (1.3-1.4)	1.4 ± 0.1	ns
Presence of risk conditions (n)	4.0 (3.0-5.0)	4.0 (3.0-5.0)	4.0 (3.0-5.0)	5.0 (4.0-5.0)	< 0.05†
High blood pressure (n/%)	27/61.3	8/72.7	19/57.5	28/56	ns
Systolic blood pressure (mmHg)	130.0 (115.0-140.0)	120.0 (120.0-150.0)	130.0 (120.0-150.0)	140.0 (124.0-150.0)	ns
Diastolic blood pressure (mmHg)	80.0 (72.0-85.0)	76 ± 10.1	80.0 (80.0-90.0)	80.0 (80.0-90.0)	ns
Fasting plasma glucose (mg/dl)	91.3 ± 5.5	92.4 ± 6.9	107 (103.5-110.0)	108.0 ± 10.0	−
Glucose tolerance test (mg/dl)	98.9 ± 22.1	149.0 (142.0-179.0)	110.5 ± 18.9	158.5 (145.0-189.5)	−
Dyslipidemia (n/%)	27/61.3	8/72.7	23/69.6	19/38	< 0.001‡
Total cholesterol (mg/dl)	212.8 ± 40.4	180.9 ± 36.9	212.5 ± 36	193.4 ± 38.7	ns
HDL-cholesterol (mg/dl)	54.5 ± 13.5	44.0 ± 12.2	52.1 ± 13.5	49.4 ± 11.3	ns
LDL-cholesterol (mg/dl)	134.4 ± 31.5	113.7 ± 35.3	133.9 ± 33.6	112.9 ± 35.8	< 0.05*,§
Triglycerides (mg/dl)	131.5 ± 73.0	115.5 ± 47.0	131.0 (93.5-156.5)	158.8 ± 81.3	ns
Uric acid (mg/dl)	5.5 ± 1.3	6.0 ± 1.1	6.4 ± 1.6	6.5 ± 1.6	< 0.05*
HOMA-IR	1.1 (0.4-2.0)	1.4 (1.0-8.3)	5.9 ± 6.3	2.6 (1.1-4.1)	< 0.05†,||

* Difference between group 1 and group 4 according to analysis of variance with Tukey's post-hoc test;

† Difference between group 1 and group 4 according to Kruskal-Wallis with Dunn's post-hoc test;

‡ Difference between group 1 and group 4 according to chi-square test;

§ Difference between group 3 and group 4 according to analysis of variance with Tukey's post-hoc test

|| Difference between group 1 and group 3 according to Kruskal-Wallis with Dunn's post-hoc test. HDL = high-density lipoprotein; LDL = low-density lipoprotein; HOMA-IR = homeostasis model assessment of insulin resistance; ns = not significant.

**Figure 2.**
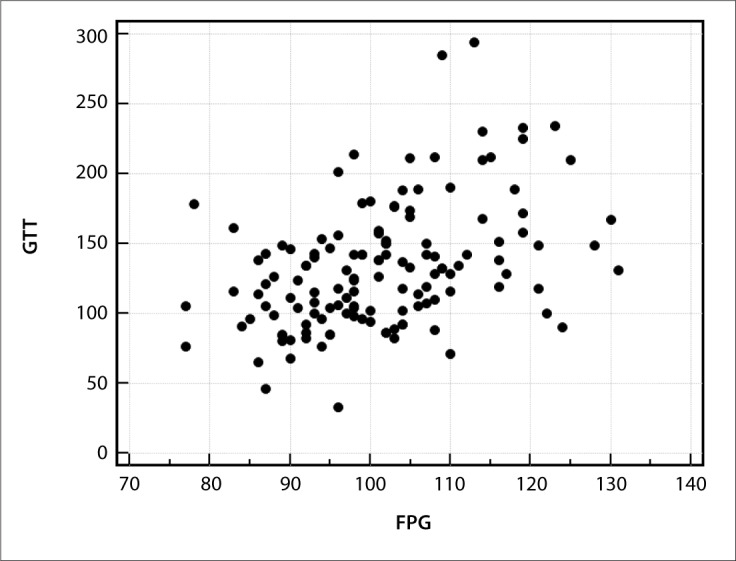
Spearman's correlation between fasting plasma glucose (FPG) and glucose tolerance test (GTT) among total sample studied (r = 0.5; P < 0.0001).

We performed correlation analysis between the variables and the GTT for the total sample (groups 1, 2, 3 and 4) and for individuals who had normal FPG (groups 1 and 2). The second analysis was performed in order to search for clinical, anthropometric or laboratory characteristics relating to glucose intolerance among individuals with normal FPG. In assessing the total study sample, FPG showed statistical correlations with GTT (r = 0.5; P < 0.0001) (Figure 2), according to Spearman's correlation test. In assessing individuals who presented normal FPG, no variables showed any correlation with the GTT.

**Figure 3.**
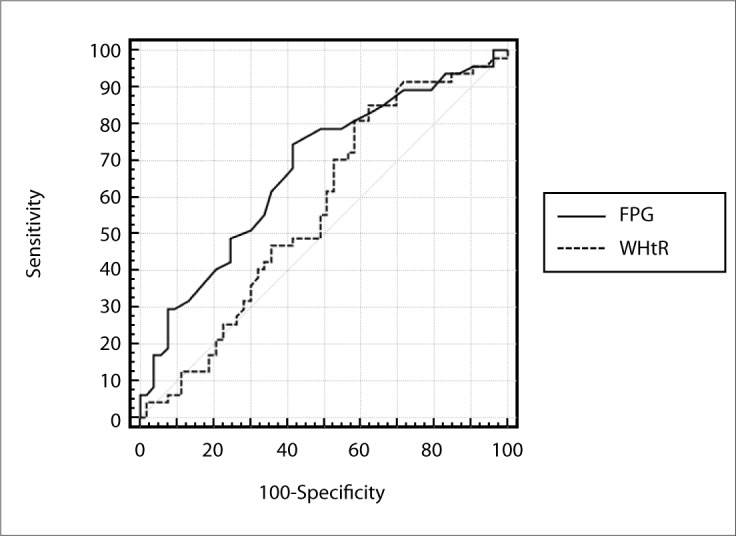
Receiver operating characteristic (ROC) curve for fasting plasma glucose (FPG) and waist-to-height ratio (WHtR) in relation to diagnosis of glucose intolerance according to glucose tolerance test (GTT) among total sample studied (n = 138).

We performed ROC analysis for FPG and anthropometric parameters in relation to glucose intolerance, diagnosed according to the GTT, in the total sample of the study (groups 1, 2, 3 and 4), and among the individuals with normal FPG (groups 1 and 2). In analyzing the total sample studied, we found that an AUC larger than the diagonal reference line (AUC > 0.5) was demonstrated by the FPG (P < 0.0001) and WHtR (P = 0.04) (Table 3 and Figure 3), unlike the other parameters analyzed. On the other hand, among the individuals with normal FPG, neither FPG nor anthropometric parameters showed areas under the ROC curve larger than the diagonal reference line (P > 0.05) (Table 4). We also evaluated the cutoff points for FPG and anthropometric variables with greater accuracy in the glucose intolerance diagnosis, seeking the variables with the highest sum between sensitivity and specificity, firstly among the total sample studied (groups 1, 2, 3 and 4) and then among individuals with normal FPG (groups 1 and 2). In the first analysis, the FPG (at 98 mg/dl) and the WHtR (at 0.6) stood out (Table 3); and in the second analysis, the FPG (at 92 mg/dl) and BMI (at 28.2 kg/m^2^) stood out (Table 4).

**Table 3.** Effectiveness of variables analyzed for diagnosing glucose intolerance in the total sample studied (n = 138), according to receiver operating characteristic (ROC) analysis

Variables	AUC SE (95% CI)	Cutoff point	Sensitivity (95% CI)	Specificity (95% CI)	Sn + Sp	P (AUC = 0.5)
Fasting plasma glucose	0.7 0.1 (0.6 to 0.8)	98	76.0 (62.4 to 86.5)	56.4 (44.7 to 67.6)	132.3	< 0.0001
Waist-to-height ratio	0.6 0.1 (0.5 to 0.7)	0.6	82.4 (69.1 to 91.6)	42.1 (30.9 to 54.0)	124.5	0.04
Waist circumference	0.6 0.1 (0.5 to 0.7)	108	32.7 (20.3 to 47.1)	84.2 (74.0 to 91.6)	116.9	ns
Conicity index	0.6 0.1 (0.5 to 0.7)	1.3	66.7 (52.1 to 79.2)	51.3 (39.6 to 63.0)	118.0	ns
Body mass index	0.6 0.1 (0.5 a 0.7)	28.0	72.6 (58.3 to 84.1)	48.7 (37.0 to 60.4)	121.2	ns
Waist-to-hip ratio	0.6 0.1 (0.5 to 0.6)	1.02	25.5 (14.3 to 39.6)	89.5 (80.3 to 95.3)	115.0	ns

AUC = area under curve; SE = standard error; CI = confidence interval; Sn = sensitivity; Sp = specificity; ns = not significant.

**Table 4.** Effectiveness of variables analyzed for diagnosing glucose intolerance among individuals with normal fasting plasma glucose (n = 55), according to receiver operating characteristic (ROC) curve analysis

Variables	AUC ± SE (95% CI)	Cutoff point	Sensitivity (95% CI)	Specificity (95% CI)	Sn + Sp	P (AUC = 0.5)
Fasting plasma glucose	0.6 ± 0.1 (0.5 to 0.7)	92	68.8 (41.3 to 89.0)	55.6 (40.0 to 70.4)	124.3	ns
Waist-to-height ratio	0.5 ± 0.1 (0.4 to 0.6)	0.6	81.3 (54.4 to 96.0)	31.8 (18.6 to 47.6)	113.1	ns
Waist circumference	0.5 ± 0.1 (0.4 to 0.6)	95	56.3 (29.9 to 80.2)	61.4 (45.5 to 75.6)	117.6	ns
Conicity index	0.5 ± 0.1 (0.4 to 0.7)	1.4	75.0 (47.6 to 92.7)	40.9 (26.3 to 56.8)	115.9	ns
Body mass index	0.6 ± 0.1 (0.4 to 0.7)	28.2	75.0 (47.6 to 92.7)	52.3 (36.7 to 67.5)	127.3	ns
Waist-to-hip ratio	0.5 ± 0.1 (0.4 to 0.6)	1.02	31.3 (11.0 to 58.7)	88.6 (75.4 to 96.2)	119.9	ns

AUC = area under curve; SE = standard error; CI = confidence interval; Sn = sensitivity; Sp = specificity; not significant.

## DISCUSSION

The high prevalence of glucose intolerance observed in this sample was mainly due to the high complexity profile of the hospital where this study was developed, thus explaining the finding of higher prevalence than seen in other studies.^9-11^ It is noteworthy that, among individuals with glucose intolerance (groups 2 and 4), 18% had normal FPG. Moreover, considering all the individuals with normal FPG (groups 1 and 2), no clinical or anthropometric variables were able to screen for glucose intolerance in a statistically significant way. However, it needs to be borne in mind that because of the small number of subjects in this study, these assessments did not distinguish between the sexes. This may have reduced the accuracy of these variables, with regard to parameters for which the cutoff differs between men and women, such as waist circumference and WHtR.^18-21^

In the total study sample, the only anthropometric variable that was shown to be effective regarding the diagnosis of glucose intolerance according to the GTT, from a statistical point of view, was the WHtR. Other research groups have reported the existence of associations between WHtR and clinical conditions such as left ventricular hypertrophy,^22^ high blood pressure,^23-25^ diabetes mellitus,^24^ and insulin resistance in eutrophic men, as assessed using the HOMA-IR index.^26^ The WHtR analysis was based on studies such as by Hsieh and Yoshinaga, who showed that individuals with similar waist circumferences but small stature showed worse metabolic profile and cardiovascular disease, as demonstrated by higher prevalence of hyperglycemia, fatty liver disease and hypertension compared with individuals of greater stature, even after adjusting for age, smoking and lipid profile. This suggests that the WHtR would be a more accurate tool in screening for metabolic consequences of visceral adipose tissue deposit.^27^ This is an anthropometric parameter that encompasses variables from simple measurement and is easy to interpret, which suggests that it might be useful within clinical practice settings.

In this study, among the individuals who had normal FPG, the prevalence of glucose intolerance was 20.0%, as demonstrated by an abnormal GTT. In a prospective study, Gabir et al. observed that the cumulative incidence of diabetes mellitus over five years, among those who had normal FPG and glucose intolerance was 5.5 times higher than among those whose FPG and GTT were both normal.^28^ These data suggest, according to these authors, that among patients who present at least one condition relating to increased risk of developing diabetes mellitus, performing the GTT is highly recommendable, even among individuals who have already been found to present normal FPG. This is because the GTT is a low-cost test that is simple to implement, easy to understand and widely available at all levels of healthcare complexity.

In the ROC analysis that was made in order to evaluate the accuracy of FPG in diagnosing glucose intolerance, among the individuals with normal FPG, a cutoff of 92 mg/dl, with sensitivity of 68.8% and specificity of 55.6%, was suggested. This value is lower than what is recommended as a cutoff point for normal FPG. In a prospective study, Tirosh et al. demonstrated that the risk of developing diabetes mellitus among young men who have normal FPG was much higher among those who initially had FPG between 91 and 99 mg/dl, i.e. classified as high normal FPG.^29^ Another research group showed the same result in a population-based study among those who had FPG ≥ 94 mg/dl.^30^ Studies have shown substantial findings suggesting that high normal FPG levels are related to increased cardiovascular, cerebrovascular and overall mortality among individuals aged 45 years and over.^31,32^ Therefore, subcategories within the normal FPG range might denote important information regarding risk assessments for several pathological conditions,^33,34^ as suggested by this study in relation to glucose intolerance.

It is important to note that the main limitation of this study was inherent to its cross-sectional design. It was not possible to determine cause and effect relationships, but rather, only associations could be reported. Another important limitation of this study was the fact that plasma HbA1c assays were not performed on the research subjects, because when this study was planned and conducted, performing plasma HbA1c assays as a screening test for non-diabetic individuals was not part of the usual recommendations. However, in January 2010, this evaluation started to be recommended as routine screening for glucose metabolism abnormalities by the American Diabetes Association.^35^ Unfortunately, it was not possible to include HbA1c evaluations in this study consequent to that recommendation, because the blood samples were no longer stored.

In the present study, the GTT proved to be an important diagnostic tool for glucose metabolism alterations, even among individuals who had normal FPG, when they showed risk conditions for developing diabetes mellitus. Since FPG as a screening test was unable to detect glucose metabolism abnormalities in 8.0% of the research subjects, it suggests, in our opinion, that the GTT is well indicated for patients who present these risk conditions, regardless of having normal FPG.

Such findings may also suggest that the individuals who had normal FPG but abnormal GTT could be at an earlier stage of glucose intolerance, which is the point at which prophylactic interventions should be adopted. Although some anthropometric, clinical and laboratory findings have been described as related to diabetes mellitus and prediabetic state, none except for GTT was able to screen for occurrences of glucose intolerance among subjects with normal FPG, in the present study.

## CONCLUSION

In the sample studied, the prevalence of abnormal glucose metabolism was as high as 68.0%. These glucose metabolism abnormalities could be described as 8.0% for normal FPG but abnormal GTT, 23.9% for abnormal FPG but normal GTT, and 36.2% for elevation in both FPG and GTT. The patients in group 4 were older and presented more risk conditions for developing diabetes mellitus than did the individuals in group 1.
